# Two-Minute Step Test as a Complement to Six-Minute Walk Test in Subjects With Treated Coronary Artery Disease

**DOI:** 10.3389/fcvm.2022.848589

**Published:** 2022-05-09

**Authors:** María José Oliveros, Pamela Seron, Claudia Román, Manuel Gálvez, Rocío Navarro, Gonzalo Latin, Tania Marileo, Juan Pablo Molina, Pablo Sepúlveda, Gabriel Nasri Marzuca-Nassr, Sergio Muñoz

**Affiliations:** ^1^Facultad de Medicina, Universidad de La Frontera, Temuco, Chile; ^2^Facultad de Medicina, Pontificia Universidad Católica de Chile, Santiago, Chile; ^3^Complejo Hospitalario San José, Santiago, Chile; ^4^Hospital Clínico Universidad de Chile, Santiago, Chile; ^5^Hospital Clínico San Borja-Arriarán, Santiago, Chile; ^6^Hospital Regional de Antofagasta, Antofagasta, Chile; ^7^Hospital San Juan de Dios, Santiago, Chile

**Keywords:** step test, 6-minutes walking test, fitness assessment, exercise test [MeSH], functional capacity, coronary artery disease

## Abstract

The 2-Minute Step Test (2MST) has been presented as an alternative to the 6-Minute Walk Test (6MWT) based on the association between the two tests in older adults; however, some authors propose that it should not be a substitute but rather a complement to the latter in the fitness evaluation. Specifically, in coronary disease, despite the potential and clinical utility of 2MST, the relationship of both tests in this population is unknown. This study aimed to determine the relationship between 6MWT and 2MST and to explore the relationship of biodemographic factors for both tests in subjects with treated coronary artery disease. For this, the 6MWT and the 2MST were applied to patients with coronary artery disease treated in 6 hospitals in Chile between May 2019 and February 2020. Additionally, lower limb strength was assessed by a chair-stand test, grip strength was assessed by a dynamometer, and physical measurements were applied. In total, 163 participants underwent both tests (average age = 58.7 ± 9.8 years; 73.6% men; 64.4% revascularized by angioplasty; 28.2% revascularized by surgery, and 7.4% treated by drugs or thrombolysis). Heart rate was higher at the end of the 6MWT, while the perception of effort was greater at the end of the 2MST. There was a weak positive correlation between the 6MWT and the 2MST in subjects with treated coronary disease (*r* = 0.28, *p* = 0.0003). While age (*r* = –0.27), weight (*r* = 0.25), height (*r* = 0.49), and strength of both lower limbs (*r* = 0.41) and grip strength (*r* = 0.53) correlated weakly or moderately to the covered distance in 6MWT, the number of steps by the 2MST correlated only weakly to height (*r* = 0.23), lower limb strength (*r* = 0.34), and grip strength (*r* = 0.34). Age, weight, height, lower limb strength, and grip strength would explain better the meters walked in the 6MWT than the steps achieved in the 2MST. With these findings, we can conclude that, in patients with treated coronary artery disease, it does not seem advisable to replace 6MWT with 2MST when it is possible to do so. Additionally, the 2MST may provide additional information in the fitness evaluation. However, the usefulness of 2MST in this population needs to be further studied.

## Introduction

The assessment of fitness components is vital for the correct prescription of exercise. Specifically, cardiorespiratory fitness generates great interest as it is directly related to cardiovascular events and all-cause mortality in adult populations (1, 2). Furthermore, it is known that its protective effect on mortality is independent of health conditions and biodemographic variables (3).

In clinical practice, different tests are used to evaluate cardiorespiratory fitness, generally requiring sophisticated equipment and treadmills or cycloergometers that are not always readily available, especially in resource-constrained settings (4). For this reason, tests based on the ability to perform daily living tasks, such as walking, are becoming more and more widespread and studied. Among them, the 6-Minute Walk Test (6MWT) (5–7) is considered simple, safe (5–7), and has been shown to be useful in candidates for cardiac rehabilitation, people with pulmonary disease, people with heart failure, those with peripheral arterial disease, and post-intensive care unit (ICU) patients (6, 8–11). Specifically, in people with coronary artery disease, a moderate to high correlation between 6MWT and maximal oxygen consumption has been described (*r* = 0.56–0.93) (11). The Heart and Soul Study conducted in a population with stable coronary heart disease reported that the distance walked on the 6MWT predicted cardiovascular events, having 4 times the rate of events in people who walked less than 420 m as compared with those who walked 545 m or more (12).

On the other hand, the 2-Minute Step Test (2MST) has been used as an alternative to the 6MWT based on the association between both tests and the time on the treadmill to 85% max heart rate reported by Rikli and Jones for an older adult population (8). This test is proposed as an option, generally when the 6MWT cannot be used, either because of structural limitations or when it is necessary to prescribe exercise to people who do not have the physical capacity or ability to ambulate. In clinical practice, the 2MWT has been gaining popularity and its usefulness has been studied in different populations, such as older adults without reference to health status or adults with heart failure, chronic kidney disease, osteoporosis, Parkinson’s disease, stroke, hypertension, depression, or Alzheimer’s disease (13). However, since the motor activity of climbing stairs may be considered more physically challenging than walking, some authors propose that it should be used as a complement rather than a substitute for other fitness assessments (14).

In this context, and given the potential and clinical utility of the 2MST in people with coronary artery disease, our study aimed to determine the relationship between 6MWT and 2MST, as well as to explore the relationship of biodemographic factors for both tests in subjects with treated coronary artery disease.

## Materials and Methods

### Study, Design, and Participants

In the context of a randomized, multi-center, non-inferiority clinical trial conducted in Chile (Hybrid Cardiac Rehabilitation Trial, HYCARET) (15), the relationship between 6MWT and 2MST was analyzed in subjects with coronary artery disease treated by medication only, thrombolysis, angioplasty, or revascularization surgery between May 2019 and February 2020. Subjects were recruited from 6 hospitals in Chile and were entered into the Cardiac Rehabilitation program of the HYCARET study, between 2 weeks and 2 months from their cardiovascular event.

The HYCARET study was approved by the corresponding Ethics Committee at the Sponsor Institution: Comité Ético Científico (CEC) of Universidad de La Frontera. This approval was considered for the study implementation in two centers. In addition, four more Ethics Committees approved the protocol and a specific informed consent for implementation in their centers was provided: CEC of Servicio de Salud Metropolitano Central, CEC of Servicio de Salud Metropolitano Norte, CEC of Hospital Clínico Universidad de Chile, and CEC of Servicio de Salud Araucanía Sur.

### Variables and Measurements

Assessments were performed prior to the start of CR by trained personnel using standardized protocols for all measurements. The main evaluations are described below, however, further details of the procedures can be found in the HYCARET study protocol (15).

The 6MWT was performed in accordance with the American Thoracic Society Statement (7). Blood pressure, heart rate, oxygen saturation, and perception of exertion were evaluated before and immediately after the test. Patients were instructed to walk as much as possible for 6 min. In case the patients required detentions during the test, they were allowed as many times as necessary, but they were encouraged to resume walking as soon as possible. The total distance covered during the test was recorded.

At least 1 day after the 6MWT was performed (7), the 2MST was performed in accordance with the Senior Fitness Test (16). All parameters were evaluated before and after the test was completed, in the same manner as for the 6MWT. To determine the height of limb elevation, the midpoint between the femorotibial joint and the anterosuperior iliac crest of each participant was identified. Instructions were delivered in a standardized manner for all participants, and upper limb support was offered to those with balance problems. To initiate the test, participants were asked to raise the left limb and then the number of times the right knee reached the mark during the 2 min was counted.

Additionally, upper and lower limb strength, waist circumference, weight, height, and other sociodemographic variables were assessed to characterize the participants and explore potential associations with fitness as measured by the 6MWT and the 2MST.

Upper and lower limb strengths were evaluated by dynamometry and by standing up from a chair test, respectively. By using a Jamar dynamometer, patients were instructed to sit on a chair with armrests with the shoulder adducted, elbow articulation flexed at a 90 degree angle, forearm in a neutral position, and wrist between 0 and 30 degrees of dorsiflexion and to perform three maximal effort trials with each hand; the highest value achieved on each limb was considered. For the standing up from chair test, participants were asked to stand up from the chair from a seated position with their arms crossed at chest height, achieving full standing, and then to sit down again as many times as possible for 30 s (16).

Weight and height were measured with the subject in a bipedal position, with little clothing, using a non-automatic mechanical column scale, SECA Model 700. Waist circumference was measured at the midpoint between the lower edge of the last rib and the iliac crest at the end of a normal exhalation, using a 7-cm non-elastic flexible tape measure. For this measurement, 2 measurements were recorded; if they differed by more than 1 cm, a third measurement was requested.

Additionally, to evaluate the hemodynamic response to both tests, blood pressure, perceived exertion, heart rate, and oxygen saturation were measured before and at the end of the 6MWT and the 2MST using automatic devices, with the subject in a seated position with the upper extremity uncovered and resting on a table at heart level (17).

### Statistical Analysis

Univariate descriptive analyses were performed to examine the distribution and frequency of meters walked during the 6MWT and steps taken during the 2MST. Measures of central tendency and dispersion were used to present continuous variables, while proportions were used to describe categorical data. Differences by sex and type of treatment received were tested with a *t*-test.

The correlation coefficient and its *p*-values were estimated to answer the main study goal of determining the relationship between 6MWT and 2MST. Sensitivity analyses were carried out to explore tendencies between men and women, between those who were treated surgically or percutaneously, and according to the performance on the 6MWT.

To quantify the level of concordance between the tests, both were equally scaled to a scale ranging from 0 to 100, and then the Bland-Altman plot was performed by plotting the difference between the two paired measurements against the mean of the two measurements.

The relationships between both tests and age, weight, height, and strength were analyzed by correlation analysis.

## Results

In total, 163 participants with treated coronary artery disease performed both tests and were therefore included in this report. The average age was 58.7 ± 9.8 years, and 76.6% of the sample were men. In terms of treatment received, 64.4% were revascularized by angioplasty, 28.2% by surgery, and 7.4% were treated by drugs or thrombolysis. More details on the characteristics of the population are available in Table 1.

**TABLE 1:** Baseline characteristics, clinical presentation, and treatment.

Characteristics	
Age (years, mean ± SD)	58.7 ± 9.8
Sex (% male)	76.7
**Educational level (%)**	
Primary education	37.4
Secondary – Upper secondary	33.1
Trade or College/University	29.5
**Occupation (%)**	
Current paid work	55.8
Homemaker	6.8
Retired	13.5
Unemployed	14.7
Other	9.2
**Cause of hospital admisión (%)**	
Unstable Angina	7.4
Acute myocardial infarction	78.5
Intervention for stable coronary artery disease	14.1
**Treatment received (%)**	
Drugs or thrombolysis	7.4
Revascularization by angioplasty	64.4
Revascularization by surgery	28.2
**Physical measures**	
Weight (kg, mean ± SD)	76.3 ± 13.1
Height (cm, mean ± SD)	162.7 ± 8.2
Lower limb strength (number, mean ± SD)	14.1 ± 4.8
Grip strength (kilos, mean ± SD)	29.8 ± 9.3

The mean 6MWT distance was 478 ± 116 m. Men walked 105 ± 20 m more than women (*p* < 0.05) and patients undergoing cardiac surgery walked on average 42 ± 20 m less than those undergoing minimally invasive revascularization (*p* < 0.05).

The mean number of steps reached during the 2MST was 72 ± 34 in 30 s, and women had a lower performance than men (women, 63 ± 23 steps; men, 75 ± 24 steps; *p* < 0.05). Regarding the treatment received, there was no difference in the number of steps achieved between the patients who underwent cardiac surgery and those who underwent minimally invasive revascularization.

**FIGURE 1:**
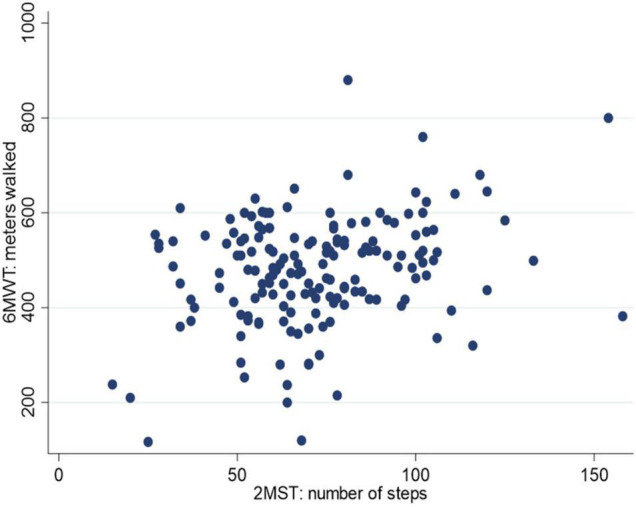
Correlation between the 2-Minute Step Test (2MST) and the 6-Minute Walk Test (6MWT).

As shown in Figure 1, the distance covered during the 6MWT and the number of steps during the 2MST were weakly correlated in subjects with treated coronary disease (*r* = 0.28, *p* = 0.0003). The sensitivity analysis showed that this correlation remained weak according to the type of treatment received (angioplasty, *r* = 0.29; cardiac surgery, *r* = 0.22) and among different sex (men, *r* = 0.2; women, *r* = 0.31). Finally, we discovered that the correlation between subjects who performed better during the 6MWT by walking 529 m or more was higher than those who walked fewer meters during the test (≤436 m *r* = 0.2903; >436 m and ≤529 m *r* = 0.11; >529 *r* = 0.45).

**FIGURE 2:**
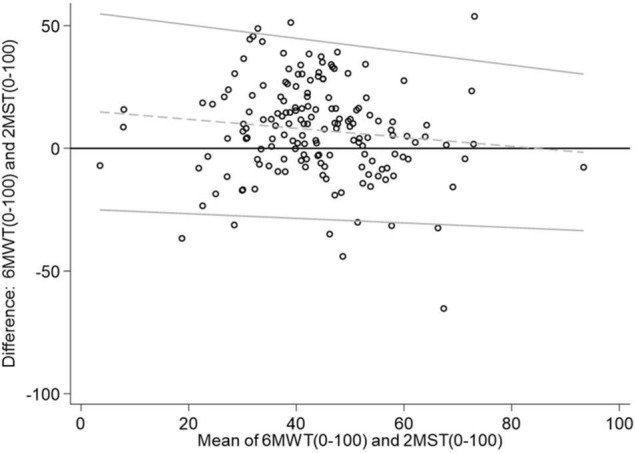
Bland–Altman plot.

Figure 2 shows that the scaled 6MWT 0–100 achieved a higher mean than the scaled 2MST, meaning that the walking test consistently achieved a higher mean than the 2MST. This difference was 21.27 in the scaled unit equivalent to 187 m walked in 6 min, which is well above a clinically significant change, indicating a possible bias in the measurements. This tendency was greater among subjects with lower functional capacity and approached zero in subjects with better performance.

On the other hand, the concordance between both tests was high, since only 6.75% of the observations lie outside the 95% *CI* limits of agreement.

Regarding the physical response to both tests, heart rate (bpm), systolic blood pressure (mm Hg), diastolic blood pressure (mm Hg), and Rating of Perceived Exertion (Borg 6–20) were higher at the end of both tests. However, this difference was greater for heart rate at the end of the 6MWT, while the perception of effort was greater at the end of the 2MST, as shown in Table 2.

**TABLE 2:** Heart rate, rating perceived exertion, and blood pressure pretests and posttests.

	6MWT			2MST			*p*-value for the difference between the changes
(mean ± SD)			Δ (mean ± SD)	(mean ± SD)		Δ (mean ± SD)	
	INITIAL	FINAL		INITIAL	FINAL		
Heart rate (bpm)	69.3 ± 11.1	100 ± 14.9	30.9 ± 13.1*	69.8 ± 10.4	94.4 ± 16.9	24.2 ± 15.9*	*p* = 0.0000
Systolic blood pressure (mm Hg)	121.5 ± 17.1	135.9 ± 20.2	14.6 ± 15.7*	116.9 ± 16.3	133.6 ± 19.8	17.6 ± 15.9*	*p* = 0.0875
Diastolic blood pressure (mm Hg)	73.5 ± 10.9	75.9 ± 11.4	2.6 ± 8.2*	72.1 ± 10.8	73.8 ± 10.9	2 ± 8.7*	*p* = 0.5221
Rating of Perceived Exertion (Borg 6–20)	8.1 ± 2.6	12.4 ± 2.8	4.4 ± 2.7*	7.4 ± 2.1	13 ± 2.8	5.6 ± 3.1*	*p* = 0.0002

(*) p < 0.05; (Δ) difference between initial and final evaluation; (SD) standard deviation.

In addition, we examined the association with other variables that have been shown to be a source of variability for the meters run in the walking test, such as age, weight, height, lower limb strength, and grip strength. For the 6MWT, age (*r* = –0.27) and weight (*r* = 0.25) correlated weakly with meters walked, while height (*r* = 0.49) and strength of both lower limbs (*r* = 0.41) and grip strength (*r* = 0.53) were moderately correlated to the meters walked in this test. On the other hand, the number of steps by 2MST correlated weakly with height (*r* = 0.23), lower limb strength (*r* = 0.34), and grip strength (*r* = 0.34), and unlike what was observed in the 6MWT, weight was not correlated to the steps achieved, which makes this test more suitable for overweight and obese patients, as shown in Table 3.

**TABLE 3:** Correlation between both tests and biodemographic variables.

	6MWT	2MST
	Overall	Overall
Age (years)	−0.27*	−0.13
Weight (kg)	0.25*	0.09
Height (cm)	0.49*	0.23*
Lower limb strength	0.41*	0.34*
Grip strength (kilos)	0.53*	0.34*

(*) p < 0.05.

## Discussion

The current study examined the relationship between 6MWT and 2MST and explored the relationship of biodemographic factors for both tests in subjects with treated cardiovascular disease. Our results show that, although there is a positive relationship between the two tests, it is weak, so the 2MST should still be considered as a complement to the 6MWT. However, in cases where the 6MWT cannot be performed, the 2MST seems to emerge as a valid option due to its high level of concordance.

Regarding the hemodynamic response to the effort of each test, our results showed that, although both tests generate physical effort, the increase in heart rate was greater at the end of the 6MWT, while the perception of effort was greater at the end of the 2MST, which is consistent with what has been found in other populations (14). This may be mainly because walking, although a habitual activity, involves displacement, requires balance, and is more global, while on the other hand, stair climbing is a stationary activity but more demanding for the lower limbs (18).

Similar experiences in other populations have reported contradictory results. Amaral et al. reported that there was no correlation between the steps achieved in the 2MST and the meters walked in the 6MWT (*r* = 0.26; *p* = 0.23) in patients with symptomatic peripheral artery disease, but that these did correlate with the number of steps in 6MWT (*r* = 0.55, *p* < 0.01) (19). In contrast, Haas et al. reported a strong correlation (*r* = 0.93) between the steps achieved in the 2MST and the meters run in the 6MWT in patients who were participating in cardiopulmonary rehabilitation (20). Along the same lines, the study by Wegrzynowska-Teodorczyk et al. performed on adults with heart failure also reported a positive but moderate correlation between both tests (*r* = 0.44; *p* = 0.0001) (14).

In this context, our results support the positive correlation between the two tests, even though it was not as strong as that reported in other populations. This could be explained by the fact that although the assessors were trained in the performance of both tests, the 2MST was considered a new assessment by the evaluator as it was not used in the context of cardiac rehabilitation programs unlike the 6MST. Additionally, the variability in the strength of the correlation between tests may be due to their submaximal nature and time limitation, which was also shown to influence the correlation with maximal oxygen consumption compared with maximal tests in patients with heart failure (21).

Our results also showed that the correlation between both tests was higher among those who walked a longer distance in the 6MWT, which could be explained by the fact that a test that provides an assessment of the patient’s ability to perform submaximal activities of daily living, such as the 6MWT, achieves a strong predictive ability of peak oxygen consumption in subjects who walked more distance (22). At this point, we consider it important to highlight that the participants included in our study were evaluated at enrollment in a cardiac rehabilitation program, therefore their poor performance could also affect the strength of the estimate.

To the best of our knowledge, our study is the first to examine the relationship between the two tests in a population with treated coronary artery disease, so we believe that our results would further our knowledge about the usefulness of both tests and will serve as a basis for future studies. However, in terms of the clinical application of these findings, we consider that we should be cautious in the interpretation and not discourage the usefulness that the 2MST can have, especially in cases where the 6MWT cannot be performed.

A limitation of our study that future studies should consider is the direct assessment of maximal oxygen consumption and the oxygen consumption during the execution of both tests, which would allow us to establish both the predictive capacity of VO2 max and explain the observed and unexplained variabilities between the two tests in this population.

Whereas our findings showed that in patients with treated coronary artery disease, the distance walked in the 6MWT and the steps performed in the 2MST are weakly correlated and that both tests correlate differently to age, weight, height, and muscle strength, we can conclude that, in patients with treated coronary artery disease, it does not seem advisable to replace 6MWT with 2MST when it is possible to do so. Additionally, the 2MST may provide additional information in the fitness evaluation. However, the usefulness of 2MST in this population needs to be further studied.

## Data Availability Statement

The raw data supporting the conclusions of this article will be made available by the authors, to those who submit a justified request.

## Ethics Statement

The studies involving human participants were reviewed and approved by Comité Ético Científico (CEC) of Universidad de La Frontera, CEC of Servicio de Salud Metropolitano Central, CEC of Servicio de Salud Metropolitano Norte, CEC of Hospital Clínico Universidad de Chile, and CEC of Servicio de Salud Araucanía Sur. The patients/participants provided their written informed consent to participate in this study.

## Author Contributions

MO conceived and designed the study, performed the statistical analysis, interpreted data, and wrote the manuscript. PSer conceived and designed the study, interpreted the data, and thoroughly reviewed the writing of the article. MG, RN, GL, TM, JM, and PSep performed the measurements and collected the data. SM reviewed the statistical analysis. All authors read and approved the final version of the manuscript.

## Conflict of Interest

The authors declare that the research was conducted in the absence of any commercial or financial relationships that could be construed as a potential conflict of interest.

## Publisher’s Note

All claims expressed in this article are solely those of the authors and do not necessarily represent those of their affiliated organizations, or those of the publisher, the editors and the reviewers. Any product that may be evaluated in this article, or claim that may be made by its manufacturer, is not guaranteed or endorsed by the publisher.
